# Frailty in Medicare Advantage Beneficiaries and Traditional Medicare Beneficiaries

**DOI:** 10.1001/jamanetworkopen.2024.31067

**Published:** 2024-08-30

**Authors:** Sandra M. Shi, Brianne Olivieri-Mui, Chan Mi Park, Stephanie Sison, Ellen P. McCarthy, Dae H. Kim

**Affiliations:** 1 Hebrew SeniorLife, Marcus Institute for Aging Research, Harvard Medical School, Boston, Massachusetts; 2 Department of Public Health and Health Sciences, Bouve College of Health Sciences, Northeastern University, Boston, Massachusetts; 3 Department of Internal Medicine, University of Massachusetts Chan Medical School, Worcester

## Abstract

**Importance:**

A growing proportion of the population is enrolling in Medicare Advantage (MA), which typically offers additional benefits compared with traditional Medicare (TM).

**Objective:**

To determine whether frailty and frailty trajectories differ between MA enrollees and TM enrollees.

**Design, Setting, and Participants:**

This retrospective cohort study used data from the National Health and Aging Trends Study (2015-2016). Analyses were conducted from August 2023 to March 2024. Participants were community-dwelling Medicare beneficiaries aged 65 years and older.

**Exposure:**

Enrollment in MA vs TM.

**Main Outcomes and Measures:**

Frailty was calculated by a frailty index (FI) (range, 0-1, with higher values indicating greater frailty) and the Fried Frailty Phenotype (FFP) score (range, 0-5, with higher values indicating greater frailty). Physical performance, including Short Physical Performance Battery (SPPB) score (range, 0-12, with higher values indicating better performance), and gait speed (meters per second) were measured. The primary outcome was the difference in FI and FFP scores from the 2015 baseline assessment to the 2016 follow-up assessment. Secondary outcomes include the 1-year changes in SPPB and gait speed.

**Results:**

The final cohort consisted of 7063 participants (2775 [23.1%] aged >80 years; 4040 [54.7%] female), representing a sample of the 38.8 million beneficiaries. There were 2583 (35.0%) MA enrollees (13.6 million) and 4480 (65.0%) TM enrollees (25.2 million). At baseline, the FI score was similar between MA and TM enrollees (mean [SD], 0.22 [0.15] vs 0.21 [0.14]), although MA enrollees had worse phenotypic frailty (496 participants [15.2%] vs 811 participants [13.7%] considered frail by FFP score), SPPB scores (mean [SD], 6.91 [3.34] vs 7.21 [3.27]), and gait speed (0.79 [0.24] m/s vs 0.82 [0.23] m/s) than TM enrollees. One year later, there were no differences between MA and TM enrollees in the 1-year change in FI score (mean [SD], 0.016 [0.071] vs 0.014 [0.066]; adjusted mean difference, 0.001 [95% CI, −0.004 to 0.005]), FFP score (mean [SD], 0.017 [1.004] vs 0.007 [0.958]; adjusted mean difference, −0.009 [95% CI, −0.067 to 0.049]), SPPB score (mean [SD], −0.144 [2.064] vs −0.211 [1.968]; adjusted mean difference, 0.068 [95% CI, −0.076 to 0.212]), and gait speed (mean [SD], −0.0160 [0.148] m/s vs −0.007 [0.148] m/s; adjusted mean difference, −0.010 m/s [95% CI, −0.067 to 0.049 m/s]).

**Conclusions and Relevance:**

In this cohort study of Medicare beneficiaries from 2015, MA enrollees experienced similar declines in frailty over 1 year compared with TM enrollees. Future work should examine whether the specific types of services covered by health insurance can impact frailty and health trajectories for older adults.

## Key Points

**Question:**

Do Medicare Advantage beneficiaries have lower levels of frailty and slower decline than traditional fee-for-service Medicare beneficiaries?

**Findings:**

In this nationally representative cohort study of 7063 community-dwelling individuals aged 65 years and older, compared with traditional fee-for-service Medicare beneficiaries, Medicare Advantage beneficiaries had higher levels of frailty at baseline but similar levels of frailty change over 1 year.

**Meaning:**

These findings suggest that enrollment in Medicare Advantage plans is not associated with altered frailty trajectories compared with Traditional Medicare, and more work is needed to better understand the health services needs of older adults with frailty.

## Teaser

This cohort study of community-dwelling Medicare beneficiaries aged 65 years and older examines whether frailty and frailty trajectories differ between Medicare Advantage and traditional Medicare enrollees.

## Introduction

In recent years, Medicare Advantage (MA) has become increasingly common, with 29.5 million enrollees, representing 51% of all Medicare beneficiaries in 2023.^1,2^ MA plans typically offer lower premiums and cover some additional benefits, such as vision screening and dental coverage, but enrollees face a more limited network of practitioners compared with traditional fee-for-service Medicare (TM).^3^ Moreover, MA beneficiaries differ from TM beneficiaries across both health and socioeconomic status,^4^ with contemporary MA enrollees more likely to be Black or Hispanic, to have lower income, and to be dually eligible for Medicaid.^5,6^ In particular, MA has special needs plans (SNPs) for dually eligible beneficiaries, who often have a higher burden of chronic or disabling conditions.

Frailty is a crucial measure of health for older adults, representing a vulnerable state of diminished physiologic reserve.^7,8^ In particular, changes in frailty over time have been associated with poor health outcomes, including hospitalization, higher health care costs, and mortality.^9,10,11^ Frailty progression may be partially mitigated by social integration and participation,^12^ which could be better supported with the additional services offered by MA plans. Although previous work^5^ has demonstrated that beneficiaries with disabilities are less likely to enroll in MA, the extent to which frailty and frailty trajectories may differ between MA and TM beneficiaries has not been examined previously. Here, we conducted a retrospective study to determine the extent to which (1) frailty and related characteristics differ between MA and TM beneficiaries, and (2) frailty and markers of physical function differ between MA and TM changes over 1 year.

## Methods

In this cohort study, we analyzed 2015 to 2016 data from the National Health and Aging Trends Study (NHATS), a nationally representative survey of Medicare beneficiaries linked to Medicare claims data. Surveys are conducted annually in participants’ homes, including medical history, socioeconomic status, cognitive and physical assessments, and functional status. For baseline comparisons of frailty status between MA and TM enrollees, we included all 7070 living community-dwelling Medicare beneficiaries in the 2015 survey, excluding 3 respondents with insufficient data to classify insurance status and 4 with insufficient data to calculate frailty. To analyze the change in frailty over time, we further excluded 305 who died, 723 who were lost to follow-up by the 2016 survey, and 27 who had insufficient data to calculate frailty (Figure).

**Figure.**
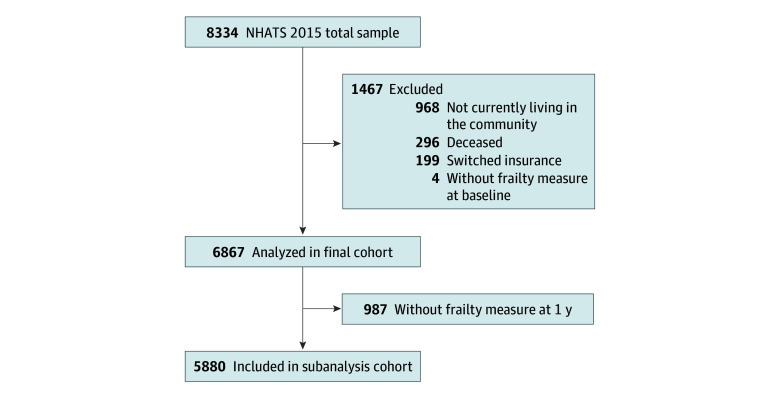
Participant Enrollment Flowchart NHATS indicates National Health and Aging Trends Study.

The Johns Hopkins institutional review board approved the NHATS protocol; all participants provided informed consent. The Advarra institutional review board approved this study of NHATS with linked Medicare data, with a waiver of consent, because the data are deidentified, in accordance with 45 CFR §46. This cohort study followed the Strengthening the Reporting of Observational Studies in Epidemiology (STROBE) reporting guidelines.

### Exposure

Our primary exposure was beneficiary insurance type classified as either MA or TM, according to Medicare enrollment status in the month of the 2015 NHATS survey. As a sensitivity analysis, we defined exposure according to 12 consecutive months of enrollment during the calendar year 2015.

### Frailty Measurements

We calculated a deficit accumulation frailty index (FI) and the Fried Frailty Phenotype (FFP) score from the 2015 survey assessment. The FI contains domains of medical history, activities of daily living, instrumental activities of daily living, physical tasks, cognitive assessments, and physical performance tests.^9,13,14^ On the basis of the FI (range, 0-1, with higher values indicating greater frailty), participants were categorized as robust (<0.15), prefrail (0.15 to <0.25), mildly frail (0.25 to <0.35), moderately frail (0.35 to <0.45), or severely frail (≥0.45). The FFP score (range, 0-5, with higher values indicating greater frailty) was defined as present on the basis of having 3 or more of the following 5 features: slow walking, weakness, exhaustion, low physical activity, and low body mass index or weight loss.^15^ The Short Physical Performance Battery (SPPB), a measure of lower extremity function and proxy of frailty, was calculated according to gait speed, chair rise, and standing balance (range, 0-12, with higher scores indicating better function).^16^ Gait speed was measured as the mean of 2 walking trials on a 3-m track, measured in meters per second.

### Outcomes

We calculated the change in frailty outcomes by assessing the change in each measure from 2015 to 2016 in FI score, FFP score, SPPB score, and gait speed. Our primary outcome was the difference in FI and FFP scores from the 2015 baseline assessment to the 2016 follow-up assessment. Secondary outcomes include the 1-year changes in gait speed and SPPB score. We also assessed the frequency with which a minimum clinically important difference (MCID) was observed. For each outcome, the MCID was defined using prior literature—specifically, an increase in 0.03 for FI,^17^ 1 for FFP,^18^ 1 for SPPB,^19^ and 0.1 m/s for gait speed.^20^ Any fall and any hospitalization in the past year were self-reported in the 2016 NHATS survey.

### Covariates

Age, sex, and race and ethnicity (Hispanic, non-Hispanic Black, non-Hispanic others, and non-Hispanic White) are self-reported in NHATS. Race and ethnicity were examined because of known differences in racial composition between our main exposure.^5^ Other race includes American Indian, Alaska Native, Asian, Native Hawaiian, and Pacific Islander. Those who answered more than 1 primary, do not know or refused to answer, or missing were treated as missing. Dual-eligible Medicare and Medicaid status was reported in the Master Beneficiary Summary File during the survey month. Annual income is self-reported in NHATS, categorized as less than $25 000, $25 000 to $50 000, $51 000 to $75 000, or greater than $75 000. Dementia was defined according to a validated NHATS algorithm, derived from subjective reports and objective cognitive testing, categorized as having no, possible, or probable dementia.^21^ Social isolation was defined as socially integrated, isolated, or severe isolation on the basis of a validated typology used in NHATS.^22^ Cohabitation status (living alone, living with a partner, living with partner and others, or living with others) and measures of social participation and engagement (eg, attending religious services or going out for enjoyment in the past month) were self-reported.

### Statistical Analysis

We compared sociodemographic and frailty measures at baseline between MA and TM beneficiaries using χ^2^ tests for categorical variables and an adjusted Wald test for continuous variables, with significance set at 2-sided *P* < .05. Linear regression was used to compare the changes (follow-up minus baseline) in gait speed and FI, FFP, and SPPB scores between MA and TM enrollees, adjusting for age, sex, dual eligibility for Medicare and Medicaid, cohabitation status, income category, and baseline scores (ie, change in FI adjusted for baseline FI). Logistic regression was used to assess the association of MA status with the likelihood of experiencing a decline by MCID in each outcome measure in 1 year. We repeated analyses in those with frailty (defined as FI ≥0.25) and with possible or probable dementia. All analyses were conducted using Stata statistical software version 15.0 (StataCorp), accounting for the complex sampling design, and were weighted to reflect national estimates.^23^ Analyses were conducted from August 2023 to March 2024.

## Results

### Characteristics of MA and TM Enrollees

Our final cohort consisted of 7063 beneficiaries, representing a sample of the 38.8 million beneficiaries. Of these, 2775 (23.1%) were older than 80 years, and 4040 (54.7%) were female. Overall, 2583 beneficiaries (35.0%) (13.6 million) were enrolled in MA, and 4480 (65.0%) (25.2 million) were enrolled in TM. MA beneficiaries were similar to TM beneficiaries in terms of age distribution (aged ≥80 years, 1011 participants [23.8%] vs 1764 participants [22.7%]) and female sex (1518 participants [56.5%] vs 2522 participants [53.7%]). MA beneficiaries were more likely than TM beneficiaries to be of Black race (640 participants [9.9%] vs 852 participants [7.8%]), Hispanic ethnicity (247 participants [12.3%] vs 184 participants [4.9%]), and other race (78 participants [4.3%] vs 130 participants [3.8%]); to have an estimated income less than $25 000 (1784 participants [65.0%] vs 2772 participants [55.6%]); and to be dually eligible for Medicare (485 participants [16.3%] vs 579 participants [10.1%]) (Table 1).

**Table 1.** Characteristics of Medicare Advantage and Traditional Medicare Enrollees

Characteristic	Participants, No. (%)			*P* value
	Medicare Advantage (n = 2583)	Traditional Medicare (n = 4480)	Overall (N = 7063)	
Population estimate, No. (weighted %)^a^	13 576 963 (65.0)	25 215 308 (65.0)	38 792 271 (100.0)	NA
Age group, y				
<80	1572 (76.2)	2716 (77.3)	4288 (76.9)	.36
≥80	1011 (23.8)	1764 (22.7)	2775 (23.1)	
Sex				
Male	1065 (43.5)	1958 (46.3)	3023 (45.3)	.05
Female	1518 (56.5)	2522 (53.7)	4040 (54.7)	
Race and ethnicity				
Hispanic	247 (12.3)	184 (4.9)	431 (7.5)	<.001
Non-Hispanic Black	640 (9.9)	852 (7.8)	1492 (8.5)	
Non-Hispanic White	1553 (73.5)	3207 (83.5)	4760 (80.0)	
Non-Hispanic other^b^	78 (4.3)	130 (3.8)	208 (4.0)	
Sociodemographic				
Speaks a non-English language	220 (20.4)	335 (16.7)	555 (17.9)	.05
Annual household income, $				
<25 000	1784 (65.0)	2772 (55.6)	4556 (58.9)	<.001
25 000-50 000	400 (15.3)	679 (15.1)	1079 (15.2)	
51 000-75 000	201 (8.9)	399 (10.7)	600 (10.0)	
>75 000	198 (10.9)	630 (18.6)	828 (15.9)	
Dual-eligible status	485 (16.3)	579 (10.1)	1064 (12.3)	<.001
Comorbidities				
No. of comorbidities, mean (SD)	3.55 (2.08)	3.56 (1.99)	3.56 (2.02)	.86
Hypertension	1855 (66.2)	3138 (65.3)	4993 (65.6)	.57
Arthritis	1584 (56.1)	2735 (56.2)	4319 (56.2)	.96
Diabetes	802 (28.5)	1195 (25.1)	1997 (26.3)	.03
Osteoporosis	630 (23.1)	1069 (21.2)	1699 (21.9)	.14
Cancer	680 (23.9)	1313 (27.6)	1993 (26.3)	.008
Heart disease	502 (17.1)	935 (17.5)	1437 (17.3)	.74
Lung disease	468 (17.2)	816 (17.0)	1284 (17.1)	.79
Heart attack	403 (13.6)	706 (13.5)	1109 (13.6)	.91
History of stroke	296 (8.8)	504 (8.8)	800 (8.8)	.97
Dementia				
No	2049 (85.9)	3624 (88.2)	5673 (87.4)	.008
Possible	298 (9.2)	482 (8.4)	780 (8.7)	
Probable	183 (5.0)	271 (3.4)	454 (4.0)	
Frailty measures				
Frailty index score, mean (SD)	0.22 (0.15)	0.21 (0.14)	0.22 (0.15)	.08
Robust	787 (39.2)	1439 (40.5)	2226 (40.1)	.02
Prefrail	722 (27.4)	1272 (28.8)	1994 (28.3)	
Mild frailty	442 (14.9)	841 (16.0)	1283 (15.6)	
Moderate frailty	269 (8.4)	399 (6.7)	668 (7.3)	
Severe frailty	363 (10.1)	529 (8.0)	892 (8.7)	
Phenotypic frailty				
Robust	832 (38.0)	1602 (42.1)	2434 (40.6)	.03
Prefrail	1255 (46.9)	2067 (44.2)	3322 (45.2)	
Frail	496 (15.2)	811 (13.7)	1307 (14.2)	
Activities of daily living disability, mean (SD), No.	0.34 (1.01)	0.32 (0.98)	0.33 (0.99)	.35
Instrumental activities of daily living disability, mean (SD), No.	1.68 (1.80)	1.57 (1.67)	1.61 (1.72)	.03
Short Physical Performance Battery score, mean (SD) (n = 6516)	6.91 (3.34)	7.20 (3.27)	7.10 (3.30)	.03
Gait speed, mean (SD), m/s (n = 6092)	0.79 (0.24)	0.82 (0.23)	0.81 (0.23)	.003
Grip strength, mean (SD), kg (n = 6064)	27.18 (10.34)	27.87 (9.93)	27.63 (10.08)	.11
Social participation				
Cohabitation status				
Living with at least 1 other person	1811 (72.8)	3048 (72.4)	4859 (72.6)	.81
Lives alone	772 (27.2)	1432 (27.6)	2204 (27.4)	<.001
Lives with partner	930 (43.3)	1868 (49.2)	2798 (47.1)	
Lives with partner and others	277 (11.3)	409 (10.2)	686 (10.6)	
Lives with others	604 (18.2)	771 (13.0)	1375 (14.8)	
Attended religious services in the past month	1516 (56.2)	2555 (54.2)	4071 (54.9)	.20
Attended other group activities in the last month	829 (34.1)	1650 (38.7)	2479 (37.1)	.003
Visiting family or friends in the last month	2170 (87.3)	3868 (89.0)	6038 (88.4)	.05
Going out for enjoyment in the last month	1889 (78.6)	3481 (82.5)	5370 (81.2)	.001
Socially integrated	1886 (74.4)	3286 (75.1)	5172 (74.9)	.90
Socially isolated	580 (21.1)	972 (20.6)	1552 (20.7)	
Severe social isolation	117 (4.5)	222 (4.4)	339 (4.4)	

Abbreviation: NA, not applicable.

^a^ Percentages are weighted to reflect national estimates.

^b^ Other race includes American Indian, Alaska Native, Asian, Native Hawaiian, and Pacific Islander. Those who answered more than 1 primary, do not know or refused to answer, or missing were treated as missing.

MA enrollees had a higher prevalence of some comorbidities than TM enrollees, such as probable dementia (183 participants [5.0%] vs 271 participants [3.4%]) and diabetes (802 participants [28.5%] vs 1195 participants [25.1%]), but a lower prevalence of cancer (680 participants [23.9%] vs 1313 participants [27.6%]). With regard to social participation, MA beneficiaries were more likely than TM beneficiaries to live with others (604 participants [18.2%] vs 771 participants [13.0%]) and less likely to report attending group activities (829 participants [34.1%] vs 1650 participants [38.7%]) or to have gone out for enjoyment in the last month (1889 participants [78.6%] vs 3481 participants [82.5%]). Otherwise, they had social integration and social engagement similar to that of TM beneficiaries (1886 participants [74.4%] vs 3286 participants [75.1%]).

### Frailty at Baseline and Change Over 1 Year

At baseline, the mean FI score was similar (MA vs TM, mean [SD], 0.22 [0.15] vs 0.21 [0.14]) (Table 1). However, MA beneficiaries had more severe frailty by FI (moderate, 269 participants [8.4%] vs 399 participants [6.7%]; severe, 363 participants [10.1%] vs 529 participants [8.0%]) and by FFP (496 participants [15.2%] vs 811 participants [13.7%]). Overall, the level of ADL disability was similar (MA vs TM, mean [SD], 0.34 [1.01] vs 0.32 [0.98]), as were measures of physical performance (SPPB score, mean [SD], 6.91 [3.34] vs 7.21 [3.27]; gait speed, 0.79 [0.24] m/s vs 0.82 [0.23] m/s).

To compare changes in frailty after 1 year, we excluded 305 decedents (2.8%) and 723 participants who were lost to follow-up (12.1%). Those who were lost to follow-up had a higher degree of frailty than those who remained in the cohort; however, they did not differ by insurance status (MA vs TM, 276 participants [12.7%] vs 447 participants [11.9%]).

For 6008 individuals who were followed-up in 2016, score changes were similar between MA and TM beneficiaries for FFP (mean [SD], 0.017 [1.004] vs 0.007 [0.958]; adjusted mean difference, −0.009 [95% CI, −0.067 to 0.049]; *P* = .73) and FI (0.016 [0.071] vs 0.014 [0.066]; adjusted mean difference, 0.001 [95% CI, −0.004 to 0.005]; *P* = .40) (Table 2). Among populations in which performance measures were available (5073 participants for SPPB and 4866 participants for gait speed), no significant differences between MA and TM enrollees were found for 1-year SPPB score change (mean [SD], −0.144 [2.064] vs −0.211 [1.968]; adjusted mean difference, 0.068 [95% CI, −0.076 to 0.212]; *P* = .40) or 1-year gait speed change (mean [SD], MA vs TM, −0.016 [0.148] m/s vs −0.007 [0.145] m/s; adjusted mean difference, −0.010 m/s [95% CI, −0.067 to 0.049 m/s]; *P* = .16). After multivariable adjustment, MA was not associated with significant changes in FI, FFP, or SPPB score or gait speed. In adjusted logistic models, MA was not associated with the odds of having an MCID in FI, FFP, or SPPB score and gait speed, as well as falls and hospitalizations.

**Table 2.** One-Year Change in Frailty and Related Characteristics Between Traditional Medicare and Medicare Advantage Beneficiaries

Variable	1-y Change			Adjusted mean difference (95% CI)
	Traditional Medicare	Medicare Advantage	Overall	
Change, mean (SD)^a^				
FFP score	0.017 (1.004)	0.007 (0.958)	0.010 (0.974)	−0.009 (−0.067 to 0.049)
FI score	0.016 (0.071)	0.014 (0.066)	0.014 (0.068)	0.001 (−0.004 to 0.005)
SPPB score (n = 5031)	−0.144 (2.064)	−0.211 (1.968)	−0.188 (2.001)	0.068 (−0.076 to 0.212)
Gait speed, m/s (n = 4829)	−0.016 (0.148)	−0.007 (0.145)	−0.010 (0.146)	−0.010 (−0.023 to 0.002)
Minimum clinically important difference, No. (%) of participants (n = 6008)^b^				
FFP score	599 (24.7)	1011 (24.7)	1610 (24.7)	0.92 (0.80 to 1.06)^c^
FI score	792 (32.6)	1305 (30.6)	2097 (31.3)	1.05 (0.91 to 1.22)^c^
SPPB score (n = 5031)	725 (39.8)	1335 (39.9)	2060 (39.9)	0.98 (0.83 to 1.15)^c^
Gait speed (n = 4829)	440 (25.1)	741 (22.7)	1181 (23.5)	1.16 (0.96 to 1.40)^c^
Any fall	688 (30.0)	1286 (32.5)	1974 (31.6)	0.87 (0.76 to 1.00)^c^
Any hospitalization	495 (20.5)	894 (21.0)	1389 (20.8)	0.89 (0.76 to 1.04)^c^

Abbreviations: FFP, Fried Frailty Phenotype; FI, frailty index; SPPB, Short Physical Performance Battery.

^a^ Change in scores are calculated as the difference from 2015 to 2016 survey measurements. For frailty scores, higher scores are worse. For SPPB and gait speed, higher scores are better. Models are adjusted for age, sex, dual eligibility for Medicare and Medicaid, cohabitation status (alone vs partner vs partner and others vs others), categorical income, and baseline status (ie, change in FI adjusted for baseline FI).

^b^ Minimum clinically important differences are 0.03 for FI, 1 for SPPB, and 0.1 m/s for gait speed.

^c^ Data are odds ratio (95% CI).

Analyses in prespecified subpopulations of persons with frailty (Table 3) and those with cognitive impairment (Table 4) yielded similar results. There were no significant differences between MA and TM populations in changes in FI, FFP, or SPPB score, gait speed, or odds of having an MCID in any of these measures. When analyses were repeated requiring 12 months of enrollment, results were similar, with no changes in 1-year frailty trajectories between the 2 populations.

**Table 3.** One-Year Change in Frailty and Related Characteristics Between Traditional Medicare and Medicare Advantage Beneficiaries With Frailty

Variable	1-y Change			Adjusted mean difference (95% CI)
	Medicare Advantage	Traditional Medicare	Overall	
Change, mean (SD)^a^				
FFP score	−0.100 (1.519)	−0.088 (1.477)	−0.092 (1.491)	0.003 (−0.112 to 0.117)
FI score	0.009 (0.107)	0.015 (0.107)	0.013 (0.107)	−0.005 (−0.014 to 0.005)
SPPB score (n = 1913)	−0.054 (2.698)	−0.130 (2.470)	−0.105 (2.549)	0.055 (−0.173 to 0.284)
Gait speed, m/s (n = 1556)	−0.016 (0.169)	−0.014 (0.165)	−0.015 (0.167)	−0.008 (−0.030 to 0.013)
Minimum clinically important difference, No. (%) of participants (n = 2542)^b^				
FFP score	289 (31.1)	494 (30.1)	783 (30.4)	1.07 (0.86 to 1.32)^c^
FI score	364 (38.2)	634 (37.4)	998 (37.7)	1.04 (0.85 to 1.28)^c^
SPPB score (n = 1913)	262 (37.6)	483 (37.4)	745 (37.5)	1.03 (0.78 to 1.37)^c^
Gait speed (n = 1556)	143 (23.8)	241 (22.7)	384 (23.1)	1.22 (0.95 to 1.57)^c^
Any fall	401 (43.5)	743 (49.4)	1144 (47.4)	0.80 (0.62 to 1.04)^c^
Any hospitalization	308 (35.0)	591 (37.4)	899 (36.6)	1.00 (0.94 to 1.06)^c^

Abbreviations: FFP, Fried Frailty Phenotype; FI, frailty index; SPPB, Short Physical Performance Battery.

^a^ Change in scores are calculated as the difference from 2015 to 2016 survey measurements. For frailty scores, higher scores are worse. For SPPB and gait speed, higher scores are better. Models are adjusted for age, sex, dual eligibility for Medicare and Medicaid, cohabitation status (alone vs partner vs partner and others vs others), categorical income, and baseline status (ie, change in FI adjusted for baseline FI).

^b^ Minimum clinically important differences are 0.03 for FI, 1 for SPPB score, and 0.1 m/s for gait speed.

^c^ Data are odds ratio (95% CI).

**Table 4.** One-Year Change in Frailty and Related Characteristics Between Traditional Medicare and Medicare Advantage Beneficiaries With Cognitive Impairment

Variable	1-y Change			Adjusted mean difference (95% CI)
	Traditional Medicare	Medicare Advantage	Overall	
Change, mean (SD)^a^				
FFP score	−0.036 (1.588)	−0.039 (1.519)	−0.037 (1.561)	−0.015 (−0.192 to 0.163)
FI score	0.028 (0.113)	0.028 (0.116)	0.028 (0.114)	0.001 (−0.012 to 0.014)
SPPB score (n = 717)	−0.231 (2.563)	−0.222 (2.710)	−0.228 (2.619)	−0.108 (−0.479 to 0.263)
Gait speed, m/s (n = 568)	−0.022 (0.178)	−0.034 (0.170)	−0.026 (0.175)	−0.016 (−0.046 to 0.014)
Minimum clinically important difference, No. (%) of participants (n = 957)^b^				
FFP score	177 (30.4)	125 (31.8)	302 (30.9)	1.04 (0.74 to 1.46)^c^
FI score	242 (39.7)	173 (44.3)	415 (41.5)	1.26 (0.89 to 1.77)^c^
SPPB score (n = 717)	189 (41.0)	112 (40.9)	301 (40.9)	1.08 (0.74 to 1.59)^c^
Gait speed (n = 568)	92 (24.5)	63 (26.1)	155 (25.1)	1.12 (0.67 to 1.88)^c^
Any fall	235 (42.7)	146 (39.8)	381 (41.6)	0.89 (0.59 to 1.34)^c^
Any hospitalization	170 (28.7)	117 (30.0)	287 (29.2)	1.04 (0.74 to 1.45)^c^

Abbreviations: FFP, Fried Frailty Phenotype; FI, frailty index; SPPB, Short Physical Performance Battery.

^a^ Change in scores are calculated as the difference from 2015 to 2016 survey measurements. For frailty scores, higher scores are worse. For SPPB and gait speed, higher scores are better. Models are adjusted for age, sex, dual eligibility for Medicare and Medicaid, cohabitation status (alone vs partner vs partner and others vs others), categorical income, and baseline status (ie, change in FI adjusted for baseline FI).

^b^ Minimum clinically important differences are 0.03 for FI, 1 for SPPB score, and 0.1 m/s for gait speed.

^c^ Data are odds ratio (95% CI).

## Discussion

In this nationally representative cohort study of Medicare beneficiaries in 2015, we compared frailty characteristics between MA and TM beneficiaries using validated frailty measures. MA beneficiaries had a higher burden of frailty by 2 validated frailty measures: FI and FFP. However, changes in frailty measures were similar in the 2 populations among beneficiaries after 1 year.

Historically, MA plans were observed to attract healthier individuals, perhaps owing to favorable selection by those who would benefit from wellness plans or were attracted to lower premiums.^24^ However in recent years, MA enrollment has grown, primarily as a result of TM beneficiaries switching to MA,^25^ rather than new enrollments, and many of those who switch are not necessarily in better health. In particular, beneficiaries who switched were more likely than new enrollees to have disabilities, possibly reflecting a need for the additional services provided.^26^ Beneficiaries may be drawn to potentially lower out-of-pocket costs or additional benefits offered by MA plans, such as vision, dental, or wellness plans.^27^ Other known characteristics associated with switching include dual-eligibility^28^ and racial or ethnic minority status, which we also found in MA beneficiaries. This suggests that the more comprehensive coverage of MA plans may be more appealing for more relatively vulnerable populations. Interestingly, despite these additional services, changes in frailty, physical performance measures, and health outcomes did not significantly differ between MA and TM populations in our study. Our study did not allow for specific examination of what supportive services were available or used at the beneficiary levels, which may vary highly from plan to plan. Thus, it remains unclear whether availability and usage of specific services may alter frailty trajectories in vulnerable populations.

Our study is observational in nature and cannot fully capture the complex reasons and motivations behind beneficiaries’ selection of insurance plans. For example, it is possible that MA beneficiaries had experienced worsening frailty before choosing MA plans, and that MA insurance may attenuate their overall health decline, thereby making their trajectories more comparable to those of TM beneficiaries. Although previous research suggests that beneficiaries with worsening function tend to switch into TM plans, this pattern may not hold for all beneficiaries, especially those who may benefit from SNPs or enhance care management and coordination. Further investigations, particularly around changes in frailty following switches in insurance plans, are needed to illuminate this issue.

Within the 1-year follow-up period, we excluded those who died or were lost to follow-up in 2016, which may have led to selection bias. Previous literature^29^ has established that mortality rates, when adjusted for age, sex, and dual-eligible status, are typically lower among MA beneficiaries. Although those who were lost to follow-up had a higher degree of frailty than those who remained in the cohort, loss to follow-up was not different by insurance status (eTable in Supplement 1). A sensitivity analysis conservatively assuming that all who were lost to follow-up experienced worsening frailty did not yield appreciably different estimates.

With the increasing role of MA plans in providing care for frail older adults, future health services research restricted to TM populations may not adequately represent the diverse, vulnerable aging population. Previous work^4^ has established that a higher proportion of low-income beneficiaries and those from racial and ethnic minoritized groups are enrolled in MA. Our study also examined social participation measures, which may be a critical modifiable factor associated with improved health outcomes.^30^ Interestingly, we found that those enrolled in MA were more likely to live with others but less likely to go out for enjoyment, possibly reflecting more limited life-space mobility,^31^ which is associated with future health care utilization.^32^ Although MA plans, specifically those for dual-eligible individuals, may cover some supplemental benefits such as meal delivery and nonmedical transportation,^33^ whether these can support or improve social participation remains unclear.

Examples of conditions targeted by SNPs include congestive heart failure and diabetes, which were also common in our study cohort. Prior work^34^ has found that MA beneficiaries not in SNPs are similar to TM beneficiaries in terms of demographics and overall chronic conditions. It is possible that MA beneficiaries in SNPs tend to have a higher degree of frailty, which is reflected in the higher prevalence of moderate-to-severe frailty seen in the MA population. However, not all MA beneficiaries with a chronic condition are necessarily enrolled in a chronic condition SNP, and we could not directly identify whether beneficiaries were specifically enrolled in these SNPs. Although at present there are SNPs specific for dementia, there was only 1 dementia SNP offered in 2014.^35^ Thus, in all likelihood, those with dementia enrolled in MA were not in a dementia SNP and may not have necessarily benefited from any dementia-specific services. Thus, our findings that MA beneficiaries were more likely to have possible or probable dementia are somewhat surprising, suggesting that those with cognitive impairments may preferentially seek MA plans regardless of SNPs.

### Limitations

Our work has limitations. First, the findings reported cannot be generalized to beneficiaries younger than 65 years, a population eligible for Medicare because of disabling conditions. Although insurance status may vary over time, we classified individuals on the basis of insurance enrollment status at the time of the survey. However, switching insurance is likely to accompany changes in health status^26^; thus, restricting our sample to those with stable insurance status would have excluded those with poorer health. Our sensitivity analysis showed similar results when we defined exposure according to 12 consecutive months of enrollment during the calendar year 2015. Our follow-up of 1 year may seem relatively short. Prior literature^17,36^ has established that changes in frailty over 1 year are associated with measurable increases in mortality risk and increased health care costs. Nonetheless, because of a high degree of loss to follow-up in later years of the NHATS survey (almost 30% by 2 years), we were unable to examine longer-term health outcomes. With longer periods of time, there is an increasing probability of switching insurance status. Although this work establishes a foundation for looking at longer-term frailty trajectories, future studies with more extended follow-up time and a larger sample size to account for time-varying exposures may shed more light on the long-term frailty trajectories. In addition, we could not link to geographic data, thus limiting our ability to examine rurality or neighborhood-level socioeconomic deprivation, which may also impact changes in frailty over time.

## Conclusions

In conclusion, in a nationally representative cohort of Medicare beneficiaries, we observed no differences in frailty or changes in frailty status between MA and TM enrollees. However, there were differences in community engagement and participation at baseline. This work builds on a growing body of literature to understand health services and delivery for frail older adults.

## Notes

Supplement 1. **eTable.** Baseline Characteristics of Respondents in the Analyzed Sample and those Excluded due to Death, Missing Frailty Measures, or Loss to Follow Up

Supplement 2.
Data Sharing Statement
