# Supplementary material for: Frailty in Medicare Advantage Beneficiaries and Traditional Medicare Beneficiaries
Source: JAMA Netw Open. 2024 Aug 30;7(8):e2431067. doi: 10.1001/jamanetworkopen.2024.31067 (PMC11365002; doi:10.1001/jamanetworkopen.2024.31067)
Supplement: Supplement 1. — eTable. Baseline Characteristics of Respondents in the Analyzed Sample and those Excluded due to Death, Missing Frailty Measures, or Loss to Follow Up [file jamanetwopen-e2431067-s001.pdf]

Supplemental Online Content

Shi S, Olivieri-Mui B, Park CM, Sison S, McCarthy EP, Kim DH. Frailty in Medicare Advantage beneficiaries and traditional Medicare beneficiaries. *JAMA Netw Open*. 2024;7(8):e2431067. doi:10.1001/jamanetworkopen.2024.31067

eTable. Baseline Characteristics of Respondents in the Analyzed Sample and those Excluded due to Death, Missing Frailty Measures, or Loss to Follow Up

This supplemental material has been provided by the authors to give readers additional information about their work.

© 2024 Shi S et al. *JAMA Network Open*.

eTable. Baseline Characteristics of Respondents in the Analyzed Sample and those Excluded due to Death, Missing Frailty Measures, or Loss to Follow Up

	Total (n=7,063) n (%)[b]	Analyzed Sample (n=6,008) n (%)[b]	Sample excluded[a] due to death, missing frailty measures, or loss to follow up. (n=1,055) n (%)[b]	p-value
Population estimate (weighted N), %[c]	38,792,271 (100.0)	32,889,154 (84.8)	5,903,117 (15.2)	
Traditional Medicare, (weighted N), %[c]	25,215,308 (100.0)	21,524,074 (85.4)	3,691,235 (14.6)	0.116
Medicare Advantage, (weighted N), %[c]	13,576,963 (100.0)	11,365,081 (83.7)	2,211,883 (16.3)	
Frailty Index				
Robust	2,226(40.1)	1,975(41.4)	251(32.8)	<0.001
Pre-frail	1,994(28.3)	1,756(29.1)	238(24.2)	
Mildly	1,283(15.6)	1,079(15.2)	204(17.8)	
Moderate	668(7.3)	548(6.9)	120(9.5)	
Severely	892(8.7)	650(7.4)	242(15.8)	
Fried Frailty Phenotype				
Robust	2,434(40.6)	2,180(42.3)	254(31.6)	<0.001
Pre-frail	3,322(45.2)	2,824(44.9)	498(46.8)	
Frail	1,307(14.2)	1,004(12.9)	303(21.6)	
Less than 80 years	4,288(76.9)	3,713(77.6)	575(73.4)	0.004
80 years and over	2,775(23.1)	2,295(22.4)	480(26.7)	
Male	3,023(45.3)	2,560(45.3)	463(45.1)	0.907
Female	4,040(54.7)	3,448(54.7)	592(54.9)	
Race/Ethnicity				
Hispanic	431(7.5)	359(7.2)	72(9.2)	<0.001
Non-Hispanic Black	1,492(8.5)	1,247(8.2)	245(10.3)	
Non-Hispanic White	4,760(80.0)	4,118(81.1)	642(73.7)	
Non-Hispanic Other[d]	208(4.0)	159(3.5)	49(6.9)	
Speak a language other than English	555(17.9)	437(17.3)	118(21.4)	0.026

© 2024 Shi S et al. *JAMA Network Open*.

Income				
**<$25k**	4,556(58.9)	3,765(56.7)	791(71.3)	<0.001
**$25-50k**	1,079(15.2)	959(15.8)	120(11.9)	
**$51-75k**	600(10.0)	542(10.7)	58(6.0)	
**>$75k**	828(15.9)	742(16.8)	86(10.8)	
**Dual eligible status**	1,064(12.3)	883(11.9)	181(14.2)	0.044

[a]Excluded sample includes 305 deceased, 723 lost to follow up, and 27 with missing frailty measurements.
[b]Presented are sample sizes (n) and weighted proportions (column %).
[c]Presented are the population estimates (N) based on weighted samples and corresponding weighted proportions (row %).
[d]Other race includes: American Indian, Alaska Native, Asian, Native Hawaiian, Pacific Islander. Those who answered more than one primary, Don’t know or refused to answer, or missing were treated as missing
