# Supplementary material for: Frailty in Medicare Advantage Beneficiaries and Traditional Medicare Beneficiaries
Source: JAMA Netw Open. 2024 Aug 30;7(8):e2431067. doi: 10.1001/jamanetworkopen.2024.31067 (PMC11365002; doi:10.1001/jamanetworkopen.2024.31067)
Supplement: Supplement 2. — Data Sharing Statement [file jamanetwopen-e2431067-s002.pdf]

# Data Sharing Statement

Shi. Frailty in Medicare Advantage Beneficiaries and Traditional Medicare Beneficiaries. *JAMA Netw Open*. Published August 30, 2024. doi:10.1001/jamanetworkopen.2024.31067

**Data**
**Data available:** No

**Additional Information**
**Explanation for why data not available:** The dataset used here is subject to Center for Medicare Services Data use agreements
